# INTEREST GROUP SESSION—NURSING CARE OF OLDER ADULTS: MAKING IT HAPPEN: PERSON-CENTERED DEMENTIA CARE FOR BEHAVIORAL AND PSYCHOLOGICAL SYMPTOMS

**DOI:** 10.1093/geroni/igz038.2060

**Published:** 2019-11-08

**Authors:** Ann M Kolanowski, Barbara Bowers

**Affiliations:** 1 Penn State, University Park, Pa., United States; 2 University of Wisconsin-Madison, School of Nursing, Madison, Wisconsin, United States

## Abstract

Person-centered care is the standard of practice in communal living sites, yet many facilities struggle to make this a reality. Both direct care staff and administrators find residents’ behavioral and psychological symptoms of dementia (BPSD) particularly challenging. Our research team is conducting a pragmatic trial, the goal of which is to help staff use person-centered, non-pharmacological approaches for these symptoms. During the past three years we have gained insights into what may affect the ability of staff to deliver high quality person-centered care. We share these insights in this symposium. In the first paper, the investigators present data indicating that a high rate of psychotropic drug use among residents with dementia persists despite little association to BPSD, and bring into question the need for education around de-prescribing practices. In the second paper, the investigators discuss the conceptual basis and empirical evidence for using affect balance, in addition to symptom reduction, as an important and meaningful outcome for residents. The third paper examines gender differences in the expression of BPSD and how these differences may work to limit staff ability to identify treatment approaches for women who, nonetheless, have significant symptoms. In the final paper the psychometric properties and results of a new Knowledge of Person-centered Approaches for BPSD Test, that was developed by the team and given to staff, are examined and the implications of the findings for the delivery of person-centered care are considered. The discussant will reflect on these findings and provide direction for future research and practice.

